# The Case of the Children's Hospitals

**Published:** 1919-02-15

**Authors:** Cooper Perry

**Affiliations:** Hon. Sec. the College of Nursing, &c., &c.


### THE EDITOR'S LETTER-BOX.

**[Correspondence on all subjects is invited, but we cannot in any way be responsible for the opinions expressed by our correspondents, who must give their name and address as a guarantee of good faith, but not necessarily for publication. Correspondents are reminded that brevity of style and conciseness of statement greatly facilitate early insertion.]**

### THE CASE OF THE CHILDREN'S HOSPITALS.

By SIR COOPER PERRY, M.D., Hon. Sec. the College of Nursing, &c., &c.

*To the Editor of* The Hospital.

Sir,—Perhaps you will allow me to say a few words in reply to the letter of the Chairman and Vice-Chairman of the Hospital for Sick Children, Great Ormond Street, which appears under the above heading in last week's issue of The Hospital.

If I wished to take up space with merely verbal criticisms, I might point out that it was Sir Arthur Stanley and not myself who was the chairman at the meeting which took place recently at the Royal Society of Medicine, and that what we were discussing there was not the "Draft Bill for the College of Nursing," but the Bill promoted by the College for the training and registration of nurses. This inaccuracy of description leads me to think that there is some confusion in the minds of your correspondents between the Voluntary Register of the College, on which are now about 12,000 names, and the Legal Register of Nurses, which both the College Bill and the rival Bill of the Central Committee seek to set up with the sanction of Parliament.

The former Register, with the exception, as Sir Arthur Stanley then put it, "of something like ten laymen and a few doctors," is by the Memorandum and Articles of Association of the College of Nursing, Limited, confined absolutely to women trained in general nursing. And general training means the nursing of men, women, and children, just as, *pace* your correspondents, a general hospital means a hospital of a certain size with beds for men, women, and children, not limited to patients suffering from particular forms of disease. As distinct from such general hospitals all others may, I venture to think, fairly be described as "special," whether the limitation is by age, as in children's hospitals, by age and sex, as in hospitals for women, or by disease, as in fever and mental hospitals. To describe children's hospitals as "general hospitals with an age limit," is to my mind ingenious rather than convincing, unless it be claimed, for example, that adult women suffer from no diseases from which the female child is exempt.

I come now to consider what the position will be when Legal Registration comes into force. Under the College Bill there will be a general Register for Women Nurses, a condition for admission to which will be not less than three years' *general* training in a nurse-training school or schools recognised by the General Nursing Council, and there will be Supplementary Registers for male nurses, and for mental nurses, and there *may be*, but only if the General Nursing Council and the Privy Council so decide, other Supplementary Registers—*e.g.*, for women trained under approved conditions in children's nursing. I am at a loss, therefore, to understand how I can be fairly described as "dismissing the children's hospitals summarily from all participation in the benefits of the Bill." The College Bill goes so far as to leave a loophole for the admission of children's nurses to a special Supplementary Register, with protection for the title of "Registered Children's Nurse" as distinct from the title of "Registered Nurse," which would be strictly confined to nurses on the General Register.

The Central Committee's Bill, on the other hand, does not even leave a loophole for the children's nurses to get on a Supplementary Register; for under it there can be only two Supplementary Registers, one for male and one for mental nurses. All other Supplementary Registers are excluded, and it surely illustrates the irony of things that the children's hospitals which were instrumental in defeating the scheme for the amalgamation of the Royal British Nurses' Association with the College should now find in the Secretary of that Association one of the strongest opponents to any Supplementary Register which would admit their nurses to the privileges of registration on any terms.

As, however, Miss Musson's speech showed, there are members of the College Council also who are reconciled to the possibility of additional Supplementary Registers in the future only by the hope that such a provision may facilitate the passing of the Bill by showing the children's hospitals and other opponents that the door is not for all time "banged and barred" against Supplementary Registers. Should it turn out that even this concession fails to conciliate opposition, the omission of the provision in question from the College Bill will bring it into accord in this respect with the Central Committee's Bill, and if the feeling shown at the Royal Society of Medicine can be taken as an index of sentiment outside, such an omission will be welcomed by the majority of nurses now in practice.—Yours truly,

February 10, 1919. E. Cooper Perry.

